# Large-scale characterization of the macrolide resistome reveals high diversity and several new pathogen-associated genes

**DOI:** 10.1099/mgen.0.000770

**Published:** 2022-01-27

**Authors:** David Lund, Nicolas Kieffer, Marcos Parras-Moltó, Stefan Ebmeyer, Fanny Berglund, Anna Johnning, D. G. Joakim Larsson, Erik Kristiansson

**Affiliations:** ^1^​ Department of Mathematical Sciences, Chalmers University of Technology and University of Gothenburg, Gothenburg, Sweden; ^2^​ Centre for Antibiotic Resistance Research (CARe), University of Gothenburg, Gothenburg, Sweden; ^3^​ Department of Infectious Diseases, Institute of Biomedicine, Sahlgrenska Academy, University of Gothenburg, Gothenburg, Sweden; ^4^​ Department of Systems and Data Analysis, Fraunhofer-Chalmers Centre, Gothenburg, Sweden

**Keywords:** antimicrobial resistance, HMM, microbiome, phylogenetics, horizontal gene transfer

## Abstract

Macrolides are broad-spectrum antibiotics used to treat a range of infections. Resistance to macrolides is often conferred by mobile resistance genes encoding Erm methyltransferases or Mph phosphotransferases. New *erm* and *mph* genes keep being discovered in clinical settings but their origins remain unknown, as is the type of macrolide resistance genes that will appear in the future. In this study, we used optimized hidden Markov models to characterize the macrolide resistome. Over 16 terabases of genomic and metagenomic data, representing a large taxonomic diversity (11 030 species) and diverse environments (1944 metagenomic samples), were searched for the presence of *erm* and *mph* genes. From this data, we predicted 28 340 macrolide resistance genes encoding 2892 unique protein sequences, which were clustered into 663 gene families (<70 % amino acid identity), of which 619 (94 %) were previously uncharacterized. This included six new resistance gene families, which were located on mobile genetic elements in pathogens. The function of ten predicted new resistance genes were experimentally validated in *Escherichia coli* using a growth assay. Among the ten tested genes, seven conferred increased resistance to erythromycin, with five genes additionally conferring increased resistance to azithromycin, showing that our models can be used to predict new functional resistance genes. Our analysis also showed that macrolide resistance genes have diverse origins and have transferred horizontally over large phylogenetic distances into human pathogens. This study expands the known macrolide resistome more than ten-fold, provides insights into its evolution, and demonstrates how computational screening can identify new resistance genes before they become a significant clinical problem.

## Data Summary

All datasets analysed in this study are publicly available and cited in Table 1 and within the text. The genes used to construct the hidden Markov models are listed in Table S1 (available in the online version of this article). Accession numbers of analysed genomes and metagenomes are listed in Table S3. The new genes predicted in this study are listed in Table S4.

**Impact Statement**

Macrolides are among the most frequently prescribed antibiotics and are important for treating numerous types of infections. Unfortunately, their usefulness is decreasing as pathogens keep acquiring new types of macrolide resistance genes. The origin of these genes has not been elucidated, however, macrolide resistance genes are known to be maintained by a wide variety of bacteria from different environments. The true amount and diversity of macrolide resistance genes are still unknown, however, along with their evolutionary history, which makes it difficult to know what genes will transfer into clinical environments in the future. In this study, we present a comprehensive overview of macrolide resistance genes and a large number of putative resistance genes that have not been previously reported. Importantly this includes six new mobile resistance genes in pathogens not yet reported in a clinical setting. Further, through phylogenetic analysis, we have obtained new insights into the evolution and mobilization of macrolide resistance genes. These findings significantly expand the known macrolide resistome and improve our understanding of the current state of macrolide resistance.

## Introduction

Macrolides are broad-spectrum antibiotics that act by inhibiting the protein synthesis through interaction with the large ribosomal subunit [1, 2]. Since their discovery in the early fifties, macrolides have seen extensive clinical use, becoming one of the most frequently prescribed types of antibiotics with applications within both human and animal medicine [3, 4]. Macrolides are primarily effective against Gram-positive bacteria (e.g. *Streptococcus pneumoniae* or *Mycoplasma genitalium* [5]) since the molecular structure of the drugs in combination with the decreased permeability of the cell wall makes them less potent against Gram-negative bacteria. However, semi-synthetic macrolides, notably azithromycin, show greater activity towards Gram-negative bacteria, which has led to macrolides also being considered as a first-line treatment for many proteobacterial pathogens (e.g. *Salmonella enterica* or *Shigella* sp. [6]) or as an alternative treatment when penicillin and fluoroquinolones are not applicable due to resistance or patient allergy [2, 3].

Resistance to macrolides is typically associated with one of three mechanisms – modification of the target ribosomal RNA, efflux or enzymatic inactivation of the drug [6] – and is often caused by mobile resistance genes, which spread to and between pathogens through horizontal gene transfer (HGT) [7, 8]. Two of the most common types of macrolide resistance genes are *erm* genes, encoding 23S rRNA methyltransferases, and *mph* genes, encoding GTP-dependent macrolide 2′-phosphotransferases [9, 10]. Erm enzymes either mono- or di-methylate position N6 of A2058 in the 23S rRNA (* Escherichia coli* nomenclature), which, through steric hindrance, prevents the macrolide from interacting with its binding site. In addition to both natural and semi-synthetic macrolides, this results in resistance to lincosamide and streptogramin B antibiotics [1]. The exact origins of Erm enzymes have not been determined, though they are hypothesized to have evolved from the KsgA family of highly conserved 16S rRNA methylases through mutations leading to a shift in the ribosomal target [11]. In contrast to Erm, Mph enzymes interact directly with the macrolide molecules by attaching a phosphate group to the 2′-OH group, which changes their biochemical structure such that they become unable to interact with the ribosomal target [12]. This means that, unlike Erm enzymes, Mph enzymes only protect against macrolides. Additionally, Mph enzymes do not have a universal substrate profile, meaning that not all variants protect equally well against all macrolides [13]. The origin of Mph enzymes is less clear, however, they are related to similar enzymes that phosphorylate aminoglycoside antibiotics, which in turn have been suggested to have an evolutionary relationship with eukaryotic protein kinases (e.g. cAMP-dependent protein kinase cAPK) based on structural similarity [14, 15]. While *erm* genes are the most abundant and diverse type of macrolide resistance genes, with 45 different genes described to date (<80% internal amino acid identity), *mph* genes are less numerous and with 15 genes described to date [16, 17].

Commensal and environmental bacteria are known to maintain a large and diverse collection of antibiotic resistance genes (ARGs) [18], including many yet uncharacterized ARGs that may be mobilized from their host and spread to other bacteria [19–21]. Indeed, new types of *erm* and *mph* genes are frequently being discovered in clinical settings, typically after they have been horizontally transferred to human pathogens [14, 22]. The presence of a large and diverse macrolide resistome has been further emphasized by recent studies that show that new *erm* and *mph* genes are ubiquitously present in many environments, including aquatic, terrestrial and the human microbiome [23–25]. It is, thus, likely that the macrolide resistance genes characterized to date only reflect a small part of the total diversity. Without better knowledge of the resistome, it will be almost impossible to predict what genes may be mobilized into pathogens in the future. In addition, none of the currently known *erm* and *mph* genes has a well-described evolutionary history, which makes their origin unclear. This hampers our ability to implement management strategies that delay, and preferably reduce, the transfer of new macrolide resistance genes into clinical settings.

In this study, we performed a systematic investigation of the macrolide resistome to characterize its size and diversity. Optimized probabilistic gene models were used to screen large volumes of genomic and metagenomic sequence data, which resulted in 28 340 identified genes, organized into 663 macrolide resistance gene families (<70 % amino acid identity), 44 of which contained previously known genes. Among the new resistance gene families, we identified genes from six families on mobile genetic elements (MGEs) in pathogenic hosts. In total, ten novel potential macrolide resistance genes were selected for experimental validation, of which seven induced a resistance phenotype when expressed in *E. coli *. Finally, we showed through phylogenetic analysis that the most clinically relevant *erm* genes were likely mobilized from a specific part of the Firmicutes taxonomic tree, while mobile *mph* genes are much more diverse with origins in multiple phyla. Our study significantly expands the known macrolide resistome, provides insights into its evolutionary history and identifies several new emerging genes that have already spread into human pathogens. The large collection of macrolide resistance genes predicted in this study will also facilitate early detection of new macrolide resistance determinants before they spread widely and become a global threat to public health.

## Methods

### Model creation and optimization

Three profile hidden Markov models (HMMs) were built to represent two major mechanisms of macrolide resistance: two models for ribosomal target modification by Erm 23S rRNA methyltransferases and one for enzymatic inactivation by Mph macrolide 2′-phosphotransferases. Each model was optimized using fARGene v0.1 [26]. Briefly, for each model, the sensitivity was estimated using leave-one-out cross-validation, and the specificity was estimated using a set of protein sequences that shared a close evolutionary relationship with the resistance determinant, while not conferring the resistance phenotype. Prior to creating the models, protein sequences representing known macrolide resistance genes of the relevant classes were acquired from NCBI GenBank, based on GenBank IDs provided by the official Tetracycline and MLS nomenclature website (accessed October 2019) [16, 27]. To avoid bias when creating the models, such that only the regions responsible for interaction with the macrolide would be considered, the sequences were clustered at 70% amino acid identity using usearch v8.0.1445 with parameters ‘-cluster_fast -id 0.7’ [28]. Afterwards, the representative centroid sequences for each cluster were subjected to multiple sequence alignment and phylogenetic analysis using the Clustal omega v1.2.4 web client [29].

As Erm sequences were shown to cluster into two distinct groups in the resulting phylogenetic tree (Fig. S1), it was decided to divide the sequences across two separate models, here denoted Erm type A (mostly sequences from Actinobacteria), and Erm type F (mostly sequences from Firmicutes). Combining all *erm* genes into a single model resulted in an overall lower sensitivity (results not shown). The models were built using ‘fargene_model_creation’ from fARGene v0.1 [26], from 16 and 12 representative protein sequences for type A and F, respectively, and a set of 19 protein sequences from the AdoMet MTase superfamily was used to estimate the specificity of both models. The third model, representing Mph macrolide 2′-phosphotransferases, was built from 13 reference sequences, and the specificity was estimated using a set of 49 sequences representing homologues of homoserine kinase II. For all models, domain score thresholds were assigned with the criteria that both sensitivity and specificity should be as high as possible, but with high specificity taking priority over high sensitivity to ensure a low false-positive rate. To further assess the false-positive rates of the models when classifying fragmented data, a simulated metagenome was generated from 1000 randomly selected genomes from NCBI RefSeq, that were shown to not contain macrolide ARGs during the initial analysis. The chosen genomes were fragmented into 10000 paired-end reads 100 bases in length using ART Illumina v2.5 [30], with parameters ‘-l 100 f 300 m 300 -qL 93 s 0 -na -p’, and the simulated metagenome was analysed with all three HMMs using fARGene v0.1.

### Resistance gene prediction and phylogenetic analysis

The gene models were used to predict macrolide resistance genes in all genomes from NCBI GenBank (downloaded October 2019) and 14 metagenomic datasets (Table 1) using fARGene v0.1. For each resistance mechanism, the predicted protein sequences and their corresponding reference sequences were clustered into gene families of 70% amino acid sequence identity using usearch v8.0.1445 with parameters ‘-cluster_fast -id 0.7’ [28]. An outgroup was added to the representative centroid sequences of each cluster, which were then aligned using mafft v7.23 [31], with default parameters. The outgroup used for the *mph* genes represented *aph(2’’*) genes (as in Pawlowski *et al*. [14]), and for the *erm* genes represented *ksgA* genes. Phylogenetic trees representing each macrolide resistance mechanism were generated from the alignments using FastTree v2.1.10 [32] using default parameters. The trees were re-rooted at the desired outgroup and visualized using the Interactive Tree of Life web client [33] and ggtree v2.0.1 [34]. From the analysis of the phylogenetic tree, it was noted that the Erm models still misclassified some KsgA sequences as Erm. These KsgA sequences could not be removed by adjusting the domain score threshold in fARGene without also discarding previously known *erm* genes, but the KsgA sequences were identifiably based on their location in the phylogenetic tree and omitted from further analysis. After analysis of the entire genomic and metagenomic dataset, the number of genes classified as KsgA totalled 0.67% of predicted ARG sequences for these profile HMMs, which was considered acceptable.

**Table 1.** Summary of predicted macrolide resistance genes and the analysed datasets. Numbers within brackets indicate the number of genomes or metagenomic samples associated with each dataset

		Erm	Erm	Mph	Mph	
Dataset	Size (nt)	Genes	Families^*a,b*^	Genes	Families^*a,b*^	Ref.
**Genomic**						
NCBI RefSeq [15,438]	6.21×10^10^	330	10/21	1107	13/59	[68]
NCBI Assembly [412,184]	1.71×10^12^	12423	29/314	14033	14/210	[69]
**Metagenomic**						
HMP [757]	4.69×10^12^	82	7/7	8	1/1	[42]
Human gut 1 [170]	1.93×10^11^	15	6/5	2	1/1	[44]
Human gut 2 [114]	1.32×10^12^	14	7/3	2	1/1	[43]
Pig gut [295]	1.74×10^12^	145	10/9	17	1/0	[45]
Wild baboon gut [48]	1.37×10^11^	0	0/0	0	0/0	[70]
Wild rhino gut [17]	6.21×10^10^	0	0/0	0	0/0	[71]
WWTP [70]	4.82×10^11^	49	6/35	8	4/4	[46]
Pune river [62]	3.91×10^11^	45	6/33	13	4/7	[47]
Tara oceans [245]	4.89×10^12^	2	0/2	1	0/1	[72]
Antarctic soil [3]	6.25×10^9^	0	0/0	0	0/0	[73]
Forest soil [36]	1.99×10^11^	6	1/5	6	3/2	[48]
Oilspill [13]	2.75×10^11^	0	0/0	0	0/0	[74]
Lake Hazen [8]	2.75×10^11^	32	0/21	0	0/0	[49]
Amazon river [106]	2.88×10^11^	0	0/0	0	0/0	[75]
**Total**	**1.67×10^13^**	13143	**30/392** ^*c*^	15197	**14/227** ^c^	

*a*, Amino acid identity <70%.

*b*, Known/new.

*c*, Non-redundant.

HMP, Human Microbiome Project; WWTP, Wastewater treatment plant.

### Experimental validation

Potential macrolide ARGs were synthesized by the GeneArt gene synthesis service provided by Thermo Fisher Scientific. The genes were amplified by PCR using primers including the SacI and XbaI restriction sites to the 5′ and 3′ extremities, respectively. The PCR fragments were digested and cloned into the l-arabinose-inducible pBADb vector – previously digested with the same restriction enzymes – using the T4 DNA ligase (Thermo Fisher). Ligation products were transformed by heat shock into chemically competent *E. coli* TOP10 (Invitrogen, Thermo Fisher Scientific). The growth behaviour of the different clones was determined using a range of concentrations (0.125–256 µg ml^−1^) of two macrolides antibiotics – erythromycin and azithromycin – using the Omnilog system (Biolog). Bacteria were grown in 96-well plates in Mueller–Hinton broth supplemented with 0.1% of l-arabinose to express the cloned ARGs and ampicillin (50 µg ml^−1^) to maintain the recombinant plasmid during the incubation. Redox dye A (Biolog) was added to the wells to detect and quantify the number of living cells during the incubation. Metabolic activity was measured every 15 min by analysing the colour change caused by the reduction of the dye. Growth curves and standard deviations were calculated from the mean of three independent experiments. To optimize sensitivity, growth fold-changes were calculated from the Omnilog signal at 15 h of incubation at 32 µg l^−1^ erythromycin and 2 µg l^−1^ azithromycin for each tested ARG (representing the highest concentrations for which growth was observed in the control strain and a fixed time where the differences in growth between the strains, in general, were clear). The estimated fold-change was based on the mean of the three independent replicates of the tested ARG, and the mean of four independent replicates of a negative control with the native pBADb plasmid without inserted ARG.

### Statistical analysis

To investigate whether different taxonomic groups were over- or under-represented among carriers of macrolide ARGs, phylum enrichment analysis was performed. All unique species that were found to carry at least one macrolide resistance gene were divided into groups based on their phylum, and whether they carried a known or a new resistance gene. A gene was classified as known if it displayed >79% amino acid identity to any known macrolide ARG, based on the established nomenclature [16]. The number of species within each group was then counted and compared to the total number of species from the same phylum represented in the database using Fisher’s exact test. A test with a *p*-value <0.001 was considered significant.

To test whether the ARGs selected for experimental validation resulted in a significant increase in growth, *p*-values were calculated using a one-sided two-sample *t*-test. This was done using the Omnilog signal of the replicates of ARG and negative control at 15 h of incubation, at 32 µg l^−1^ erythromycin and 2 µg l^−1^ azithromycin for each tested ARG.

### Genetic context analysis

Genomes from NCBI GenBank that were found to contain new macrolide ARGs of high interest were downloaded and a region of up to 20 kb upstream and downstream of each ARG was retrieved and annotated using GEnView [35]. Sequences that were either indicated to represent mobile genetic elements or annotated as hypothetical proteins were searched using ISFinder (accessed Dec 2020) [36] and NCBI blastx v2.11.0 [37]. Further, all retrieved genetic regions were translated in all six reading frames using emboss Transeq v6.5.7.0 [29] and analysed with 22 profile HMMs designed to identify conjugation systems, acquired from the MacSyfinder CONJScan v2.0 module [38], using hmmer v3.1b2 [39]. Finally, the full contigs in which these ARGs were found were obtained from NCBI GenBank [27] and analysed using ResFinder v4.0 [40] to identify co-localized ARGs. To identify plasmid-types associated with specific resistance genes, contigs carrying these were obtained from NCBI and analysed using PlasmidFinder v2.0 [41].

## Results

### Optimization of gene models for identification of new macrolide resistance genes

We used fARGene, a software that identifies ARGs by utilizing optimized HMMs, to identify known and new macrolide ARGs in genomic and metagenomic data [26]. Three gene models were created, representing two of the most common macrolide resistance mechanisms: ribosomal target modification by Erm 23S rRNA methyltransferases and drug inactivation through phosphorylation by Mph macrolide 2′-phosphotransferases. As Erm 23S rRNA methyltransferases showed an inherently high sequence diversity, two separate models were built to ensure high performance (Fig. S1, Table S1). Each model was optimized based on a positive dataset containing experimentally validated protein sequences and a negative dataset containing protein sequences that were evolutionarily close to the ARGs without conferring a resistance phenotype. For the Erm models, the negative sequences consisted of proteins from the AdoMet MTase superfamily, while the negative data for the Mph model consisted of sequences representing homoserine kinase II. The models displayed an overall high sensitivity for full-length genes (0.94, 1.0 and 1.0 for Erm type A, Erm type F and Mph, respectively) while the specificity was 1.0 for all models (Fig. S2, Table S2). The performance for classification of metagenomic fragments varied more, with corresponding sensitivities of 0.7668, 0.8058 and 0.9663, and specificities of 0.90504, 0.9436 and 0.9857 (Fig. S2, Table S2). For additional evaluation of the specificity, simulated metagenomic reads generated from 1000 randomly selected genomes that did not contain macrolide ARGs were used. This resulted in a measured specificity of 0.9998, 0.9998 and 0.9999, respectively.

### Identification of macrolide resistance genes in genomic and metagenomic data

Next, fARGene was applied to a large collection of genomic and metagenomic data (Table S3). From 427622 genomes retrieved from the NCBI GenBank database [27], 12753 *erm* genes (1281 unique protein sequences after clustering at 100% amino acid identity) and 15140 *mph* genes (1406 unique protein sequences) were predicted (Table 1). Analysis of 15 terabases of metagenomic data resulted in the identification of an additional 390 *erm* genes (229 unique protein sequences, 183 of which were not found in the analysed genomes) and 57 *mph* genes (31 unique protein sequences, 22 of which were not found in the analysed genomes). Of the 28340 total predicted macrolide resistance gene sequences, the 13143 *erm* sequences clustered into 422 gene families (<70% amino acid identity) of which 392 (93%) were not previously characterized. The *erm* genes predicted in metagenomic data were distributed across 114 families, and 57 new gene families exclusively contained sequences reconstructed from metagenomes. Analogously, the 15197 sequences representing *mph* genes clustered into 241 gene families of which 227 (94%) were not previously characterized (Table 1). The *mph* genes predicted in metagenomic data were distributed across 22 families, and seven new gene families exclusively contained genes reconstructed from metagenomes.

**Fig. 1.**
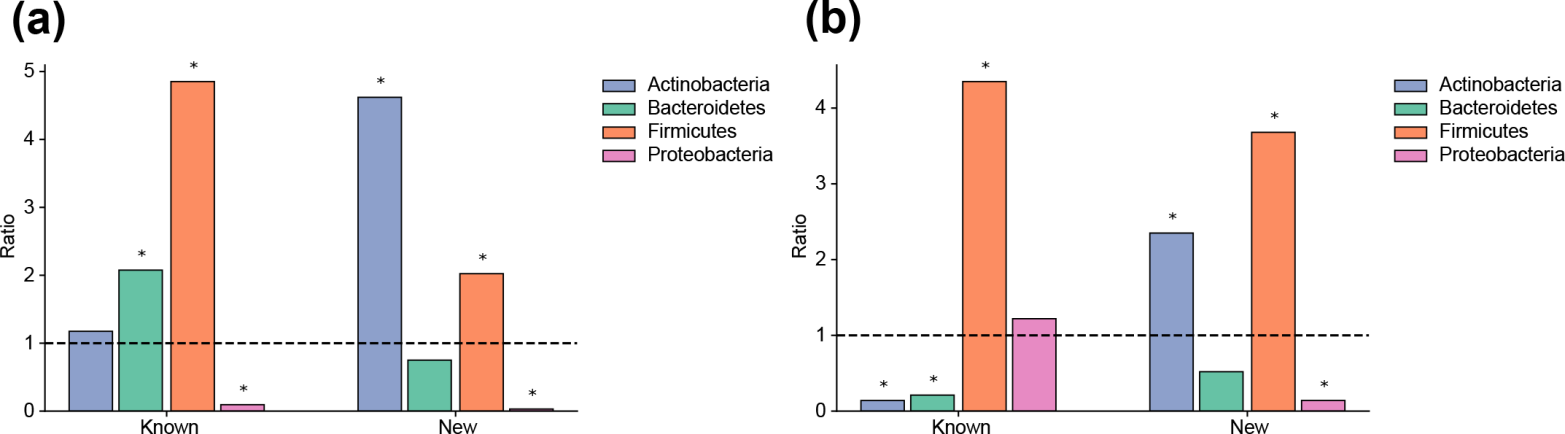
Enrichment analysis of bacterial phyla harbouring an over- or under-representation of macrolide resistance genes. The ratios and their significance were calculated using Fisher’s exact test and a star is used to denote significant results (*p*<0.001). (a) Odds ratios of known and new *erm* genes. (b) Odds ratios of known and new *mph* genes.

Of the analysed genomes, 12689 (2.97%) contained at least one *erm* gene, while 15056 genomes (3.52%) were carrying at least one *mph* gene (Table 2). Furthermore, *erm* genes could be found in 884 (6.94%) of the analysed species while *mph* genes were slightly less common, being found in 573 (4.50%) species. Enrichment analysis showed strong associations between taxonomy and the presence of *erm* and *mph* genes (Fig. 1). Firmicutes were significantly overrepresented among hosts carrying *erm* and *mph* genes, including both known (*p*<10^−15^ and *p*<10^−15^, Fisher’s exact test) and new (*p*=2.97×10^−11^ and *p*<10^−15^) variants (Fig. 1). Proteobacteria were, on the other hand, significantly under-represented among hosts of known and new *erm* genes (*p*<10^−15^ and *p*<10^−15^) as well as new *mph* genes (*p*<10^−15^). Interestingly, Actinobacteria showed a strong overrepresentation of both new *erm* and *mph* genes (*p*<10^−15^ and *p*<10^−15^) while known *mph* genes were under-represented (*p*<10^−15^). Bacteroidetes showed an overrepresentation of known *erm* genes (*p*=1.39×10^−8^) while the number of known *mph* genes was significantly lower than expected and thus under-represented (*p*=2.28×10^−6^).

**Table 2.** Proportions of the 427622 genomes and 12742 unique species in the NCBI database that carried macrolide ARGs

	Genomes (%)	Species (%)
**Erm**		
Known	2.64	3.63
New	0.33	3.67
Total	2.97	6.94
**Mph**		
Known	3.19	1.81
New	0.33	2.81
Total	3.52	4.50

**Fig. 2.**
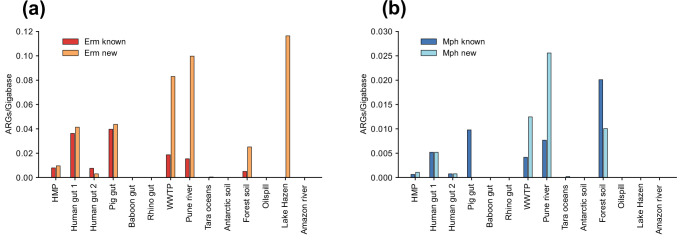
The number of reconstructed full-length macrolide ARGs per gigabase for each metagenomic dataset, divided between new and known genes. (a) Reconstructed Erm 23S rRNA methyltransferases per gigabase. (b) Reconstructed Mph macrolide 2'-phosphotransferases per gigabase. Abbreviations: HMP: Human Microbiome Project, WWTP: Wastewater treatment plant.

Analysis of metagenomic data showed that the highest number of known *erm* and *mph* genes were found in the gut microbiome of humans [42–44] and pigs [45] followed by wastewater treatment plants (WWTP) [46] and the polluted Pune river in India (Fig. 2) [47]. Interestingly, most of these environments also contained substantial levels of new genes that, in some cases, were considerably higher than those for known genes. Most of the metagenomes from non-polluted environments resulted in few or no reconstructed macrolide resistance genes, one exception being the metagenomes from soil sampled in forests in eastern China [48], which displayed high levels of both *erm* and *mph* genes. Another exception was the water samples from Lake Hazen (Canada) [49], which contained high levels of new *erm* genes but no known *erm* genes or any *mph* genes (Fig. 2).

### Experimental validation of predicted genes

The function of predicted genes was validated by expressing ten selected genes (seven *erm* genes and three *mph* genes) in an *E. coli* host and assessing the induced phenotype by observing the growth curves (Table 3, Figs S3 and S4). These genes were selected based on host species, similarity to known ARGs, and likelihood of having undergone HGT as indicated by the phylogenetic analysis. Of the ten tested genes, seven resulted in a significant increase in growth in the presence of erythromycin: *erm* genes G351, G423, G612 and G1525, as well as *mph* genes G373, G1169 and G879. The largest effect was seen for the *erm* gene G351 from the family UGF35, which displayed a growth fold-change increase of 3.4 after 15 h cultivation with 32 µg ml^−1^ erythromycin compared to the negative control. Of the seven validated genes, five genes also showed a significant increase in growth in the presence of azithromycin compared to the controls. Here, the *mph* gene G1169 from the family UGF100 showed the largest difference in growth, with a fold-change increase of 4.3 after 15 h cultivation with 2 µg ml^−1^ azithromycin. Of the ten genes, three did not show any induced phenotype in *E. coli: erm* genes G752, G883 and G1415. Note, however, that these genes might still be functional in their native or other hosts.

**Table 3.** Descriptions of identified, previously unknown macrolide resistance genes of high interest

Family [genes]	Closest known homologue [amino acid sequence identity]	Mean fold-change erythromycin (32 µg ml^−1^)	Mean fold-change azithromycin (2 µg ml^−1^)	Tested gene	Host phylum	Pathogenic host(s)	Associated MGE(s) [no. of isolates]	Co-localized ARG(s) [no. of isolates]
**Erm**								
UGF311 [4]	Erm(30) [44.7–45.2 %]	3.2*	3.8*	G1525 (* Pseudomonas aeruginosa *)	Proteobacteria	* P. aeruginosa *	IS*Xca1*-like [3], MOB_H_ [3], *tfc19* [3], IS*15DII* [2], IS*883*-like [2], IS*Ppu17* [2], *tni* transposition module [1]	*aac(6’)−29a* [2]*, aac(6’)−29b* [2]
								
UGF171 [371]	Erm(F) [59.5–64.3 %]	*NA**^a^***	*NA**^a^***	G883 (* Bacteroides fragilis *)	Bacteroidetes, Verrucomicrobia	* B. fragilis *, *Porphyromonas gingivalis *, *Prevotella intermedia *	MPF_B_ [69], MOB_V_ [52], IS*4351*-like [29], T4CP [16], MOB_P_ [11]	*tet(Q*) [269], *cfxA3* [9], *bla_OXA-347_* [1]
								
UGF246 [7]	Erm(A) [62.3–62.8 %]	1.0	1.2	G1415 (* Tetragenococcus halophilus *)	Firmicutes	–	–	–
								
UGF90 [3]	Erm(A) [60.1–62.1 %]	2.8*	3.0*	G612 (* Oceanobacillus sojae *)	Firmicutes	–	–	–
								
UGF35 [4]	Erm(T) [51.8–52.7 %]	3.4*	4.0*	G351 (* Facklamia ignava *)	Firmicutes	* Facklamia hominis *, *F. ignava *	*mobC* [2]	–
								
								
UGF46 [18]	Erm(42) [45.7–50.0 %]	2.3*	1.8	G423 (* Escherichia coli *)	Proteobacteria	* Enterobacter hormaechei *, *E. coli *, *K. pneumoniae *, *Providencia rettgeri *	Integrase (Int1) [17], IS*CR2* [10], IS*15DII* [3], IS*6100* [3], MOB_F_ [1], MOB_Q_ [2], MPF_T_ [1], IS*1B* [1], IS*4321R* [1], IS*Aba14* [1], IS*Sen9* [1]	*sul2* [10]*, sul1* [3]*, aph(3’’)-Ib* [2]*, aph(6)-Id* [2]*, bla_NDM-1_* [2]*, catA1* [2]*, dfrA1* [2]*, rmtG* [2]*, tet(B*) [2]*, aac(6’)−29a* [1]*, aac(6’)-Ian* [1]*, aadA10* [1], *aadA2b* [1]*, aadA5* [1]*, aph(3’)-VI* [1]*, armA* [1]*, bla_TEM-1B_* [1]*, bla_TEM-1C_* [1]*, bla_CARB-2_* [1]*, catB3* [1], *mph(E*) [1]*, msr(E*) [1]
								
UGF122 [12]	Erm(42) [47.0–50.3 %]	1.0	1.1	G752 (* Klebsiella pneumoniae *)	Proteobacteria	* K. pneumoniae *, * Proteus mirabilis *, * P. aeruginosa *,	*virB4* [2], IS*CR2* [2]	*sul2* [2]
								
UGF20 [28]	Erm(53) [66.7–67.5 %]	–	–	–	Firmicutes, Proteobacteria	–	MOB_Q_ [2], MPF_FATA_ [1], MPF_FA_ [1]	*tet(44*) [1]
**Mph**								
UGF5 [15]	Mph(E) [59.9–61.8 %]	3.4*	4.0*	G373 (* E. coli *)	Proteobacteria, Bacteroidetes	* E. coli *, *Salmonella enterica *	IS*CR2* [4], IS*15DII* [3], IS*15* [2], IS*Sen9* [1], *virB4* [1]	*sul2* [3]*, aadA22* [1]*, bla_CMY-2_* [1]*, bla_TEM-1B_* [1]*, erm(B*) [1]*, floR* [1]*, lnu(G*) [1], *qnrS* [1]*, tet(X4*) [1]
UGF100 [25]	Mph(O) [47.9–50.2 %]	3.2*	4.3*	G1169 (* Myxococcus xanthus *)	Proteobacteria	–	–	–
UGF37 [5]	Mph(B) [64.3–67.2 %]	1.8*	1.9	G879 (* Sporomusa termitida *)	Firmicutes	–	IS*3*-like [1]	–

*a*, Tested without replicates

*Significant increase in growth (*p*<0.001)

### Phylogenetic analysis

**Fig. 3.**
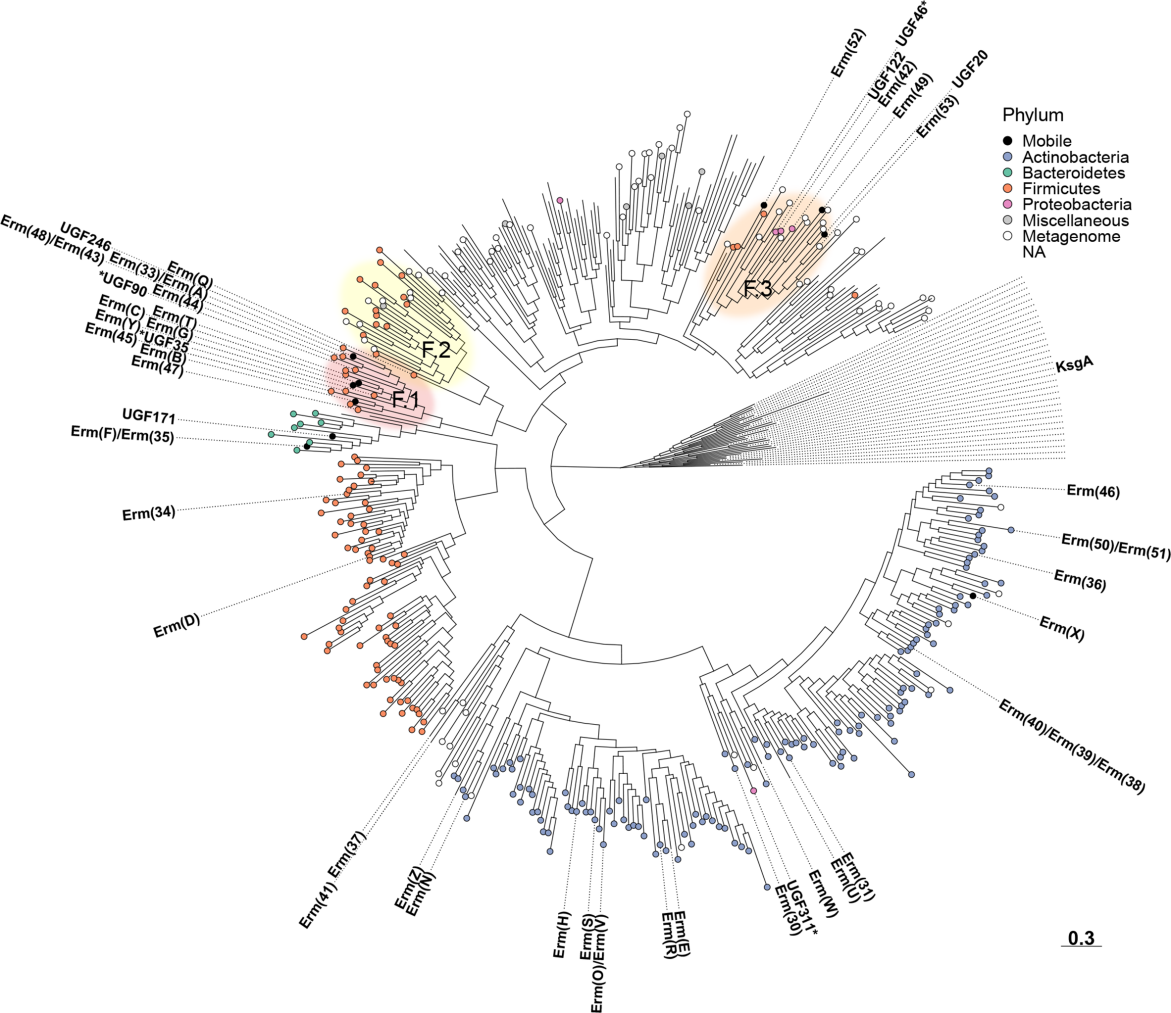
Phylogenetic tree depicting the Erm 23S rRNA methyltransferases predicted in this study. Known ARGs and new gene families of high interest are annotated in the tree and experimentally validated new ARGs are marked by a star. Each leaf is coloured based on the phylum of the identified host(s), whether it was found only in metagenomic data, or if it was discovered in multiple phyla (mobile). The tree scale is displayed at the bottom right of the figure. Additional details, including bootstrap support values, can be found in Fig. S5.

A phylogenetic tree was derived from the representative centroid protein sequences of the 422 identified *erm* gene families (Figs 3 and S5). The structure of the tree showed that the genes were divided into groups based on the taxonomy of their hosts. In particular, genes found in Actinobacteria formed a large clade, where a total of 20 previously known *erm* genes could be found. This clade also contained a previously unknown gene family (UGF311) that was identified in *Pseudomonas aeruginosa *, indicating HGT from Actinobacteria to Proteobacteria. The genes in this family were located close to genes involved in plasmid conjugation (MOB_H_ *, tfc19*), and induced a resistance phenotype when expressed in *E. coli *. Further, 67 % of contigs containing genes from UGF 311 were found to also contain aminoglycoside resistance genes [*aac(6’)−29a*, *aac(6’)−29b*] (Table 3).

The *erm* genes found in Firmicutes were split between three clades. The first clade included two known *erm* genes [*erm(D*), *erm(34*)] and contained new genes mainly found in Bacillaceae and Paenibacillaceae. This clade also contained a small cluster of genes found in Bacteroidetes, which, in addition to several new families, also contained two known *erm* genes [*erm(F*), *erm(35*)]. Here, the new family UGF171 was found in pathogenic and non-pathogenic species from Bacteroidetes as well as in a species from the Verrucomicrobia phylum (* Akkermansia muciniphila *). Almost one fifth (19%) of the genes from UGF171 were, furthermore, found close to genes involved in plasmid conjugation (MOB_Q_, MOB_P_, MPF_B_, and/or T4CP), suggesting mobility. In addition, 73% of the contigs containing a gene from UGF171 also contained genes conferring resistance to tetracycline [*tet(Q*)] and/or beta-lactam (*cfxA3*, *bla_OXA-347_*) antibiotics. Expression of a gene from this family in *E. coli* did, however, not result in a measurable resistance phenotype (Table 3).

The second Firmicutes clade could be further divided into two clusters, here denoted F.1 and F.2 (Fig. 3), which contained strikingly different numbers of mobile genes. Cluster F.1 contained as many as 12 known *erm* genes, including *erm(A*), *erm(B*) and *erm(C*) which have all previously been described in multiple phyla [16]. Most of the genes in this cluster were associated with the Bacilli class, suggesting that this may be the origin of many of the most widespread *erm* genes. More specifically, the genes most closely related to *erm(B*) were associated with the Lactobacillales order, while the genes most closely related to *erm(A*) or *erm(C*) were associated with Bacillales. In addition to the known genes, cluster F.1 also contained five previously unknown gene families, of which three genes were expressed in *E. coli* and two (UGF35 and UGF90) induced a macrolide resistance phenotype (Table 3). UGF35 was identified in pathogenic species from the genus *Facklamia* while UGF90, which shared a relatively high sequence identity with Erm(A) (60.1–62.1% amino acid identity), was identified in species from the genus *Oceanobacillus *. In clear contrast to F.1, cluster F.2 only contained a single known *erm* gene [*erm(Q*)] and the new genes were primarily found in the class Clostridia or reconstructed from gut and wastewater metagenomes. Notably, several of the mobile genes in F.1 were also found in Clostridia, suggesting it may be more advantageous for Clostridia to acquire these genes rather than the more evolutionary close genes in cluster F.2.

**Fig. 4.**
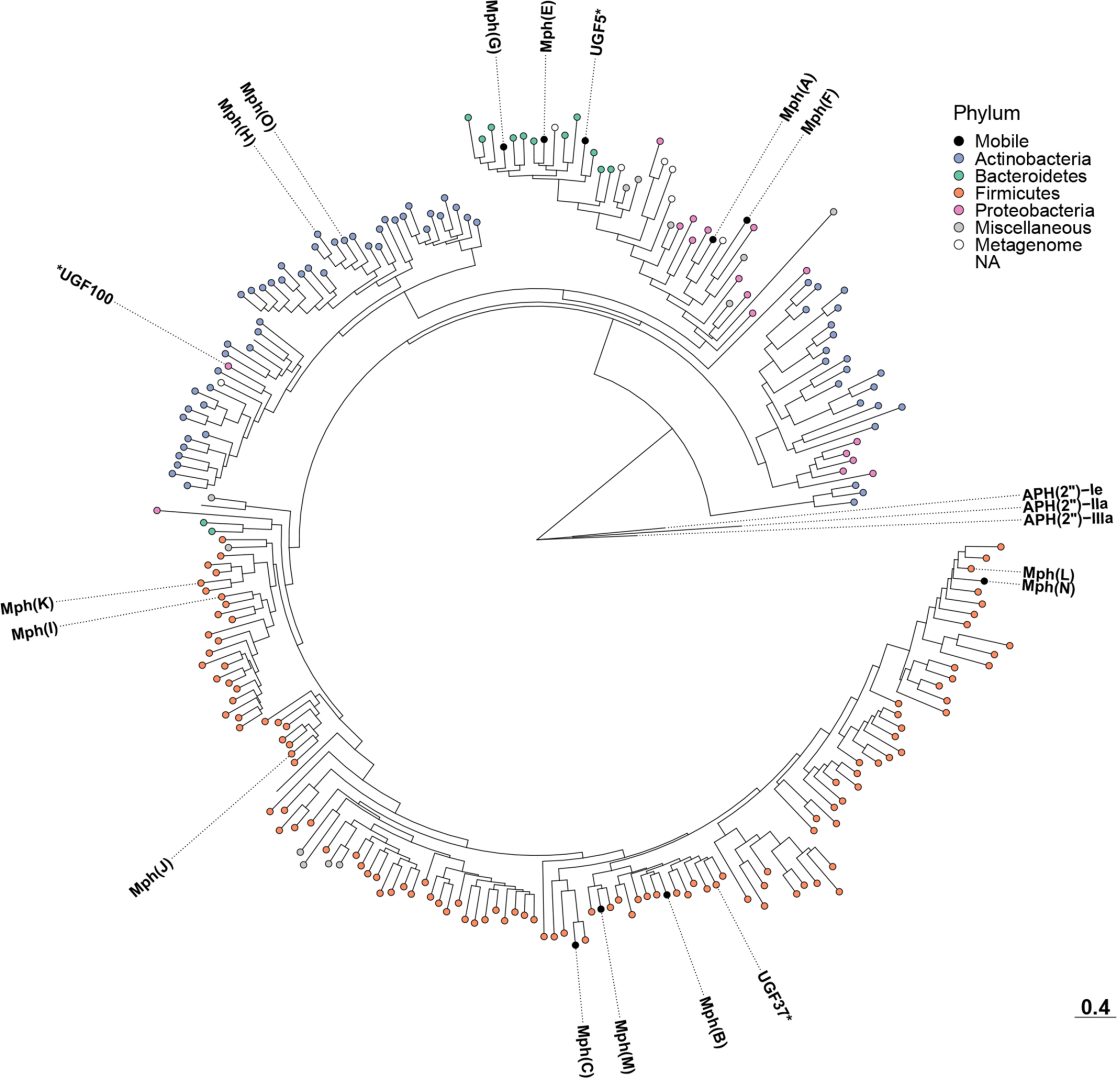
Phylogenetic tree depicting the Mph macrolide 2'-phosphotransferases predicted in this study. Known ARGs and new gene families of high interest are annotated in the tree and experimentally validated new ARGs are marked by a star. Each leaf is coloured based on the phylum of the identified host(s), whether it was found only in metagenomic data, or if it was discovered in multiple phyla (mobile). The tree scale is displayed at the bottom right of the figure. Additional details, including bootstrap support values, can be found in Fig. S6.

The final clade in the tree mostly represented unknown genes that were identified in various Candidatus phyla or reconstructed from metagenomic samples, indicating a large diversity of *erm* genes in unculturable bacteria. However, the clade also contained a small cluster, here denoted cluster F.3 (Fig. 3), that contained four known genes in addition to several unknown gene families, two of which (UGF46, UGF122) were potentially mobile and found in pathogens (Table 3). Gene families UGF46 and UGF122 were both found in Enterobacteriaceae, i.e. *Klebsiella pneumoniae *, however, when expressed in *E. coli* only UGF46 provided a resistance phenotype. Furthermore, 61% of the genes from UGF46 were found in the genetic vicinity of genes involved in conjugation (MOB_F_, MOB_Q_, MPF_T_), and/or IS*CR* elements (IS*CR2*). Notably, contigs containing genes from UGF46 were frequently found to contain other ARGs, with 55% of the contigs containing the sulfonamide resistance gene *sul2*, and 28% additionally containing one or more genes conferring resistance to other antibiotics [e.g. *msr(E)*, *aph(3’’)-Ib*, *tet(B*), *bla_TEM-1_*] (Table 3).

A phylogenetic tree was also created from the representative protein sequences of the 241 identified *mph* gene families (Figs 4 and S6). The tree consisted of four main clades, representing the taxonomy of the identified host species. The largest of these clades contained genes identified in Firmicutes and included eight known *mph* genes. This included the widespread *mph(B*) – that is commonly encountered in Enterobacteriaceae – which was located close to genes from Bacillaceae. The next clade represented *mph* genes identified in Actinobacteria and included two known genes *mph(H*) and *mph(O*). This clade contained one new gene family (UGF100) wherein all genes were identified in predatory Proteobacteria (e.g. *Myxococcus* spp.), suggesting HGT between the two phyla. A gene from this family was also found to be functional when expressed in *E. coli* (Table 3).

The next clade in the tree represented both genes from Bacteroidetes and genes from Proteobacteria. The genes associated with Bacteroidetes included two known variants [*mph(E*), *mph(G*)] as well as a new gene family (UGF5) that we were able to validate experimentally. In total, 27% of the genes from this family were flanked by IS*CR2* elements, suggesting mobility. Furthermore, 27% of the contigs containing these genes (with a large but not complete overlap with the previously mentioned subset) were found to also contain other ARGs [e.g. *erm(B*), *tet(X4*), *sul2*] (Table 3). Genes from UGF5 as well as the known *mph(E*) and *mph(G*) were found in Proteobacterial pathogens, which suggests multiple gene transfer events from this part of the tree. The final part of the tree contained genes identified in proteobacterial hosts, including the known genes *mph(A*) and *mph(F*), as well as several new genes identified in Actinobacteria.

## Discussion

In this study, we analysed over 16 terabases of bacterial DNA sequences and predicted 1614 previously unknown *erm* genes (841 unique protein sequences) across 392 gene families, as well as 1428 previously unknown *mph* genes (847 unique protein sequences) across 227 gene families (Table S4). Considering that only 44 predicted gene families (6.63%) contained previously described *erm* and *mph* genes, our findings expand the known macrolide resistome more than tenfold and thus provide a more accurate description of its size and diversity [16]. Particularly, our results show that previously unknown *erm* and *mph* ARGs are especially common in Firmicutes and Actinobacteria, while their frequency in Proteobacteria and Bacteroidetes is low in relation to their presence in the sequence repositories. Furthermore, the analysis of metagenomic data indicated that there is a large diversity of *erm* and *mph* resistance genes in the microbiome of both humans and domesticated animals. This shows that commensal bacteria – especially Firmicutes and Actinobacteria – contain a large reservoir of macrolide resistance genes that can be mobilized and spread [50]. It should also be emphasized that many new genes were found in environmental metagenomes and in unculturable bacterial species (especially *erm* genes), which are significantly under-represented in current genome databases, suggesting that the total diversity of the macrolide resistome is likely considerably larger than outlined in this study.

Genes from six new families were found to be localized on MGEs in pathogens. When expressing these genes in *E. coli *, four of six were shown to induce a resistance phenotype, thus validating that they are functional and can provide increased macrolide resistance in Proteobacteria. It should be emphasized that the two genes that did not result in a resistance phenotype could be functional in other hosts. Indeed, we were unable to validate the resistance phenotype for the new G883 (UGF171), which was related to *erm(F*), a gene that is functional in, e.g. *Bacteroides fragilis* but has previously been reported to not be functional in *E. coli* (though conflicting reports exist) [51, 52]. Furthermore, several of the new pathogen-associated mobile genes were found in Enterobacteriaceae or *Pseudomonas *, which are intrinsically resistant to lower concentrations of most macrolides [53]. This suggests that the genes have either been promoted in these pathogens under high macrolide selection pressures or that the genes were co-selected together with other ARGs, possibly through co-localization on MGEs. The latter is supported by the observation that genes from all these families were found on contigs together with genes conferring resistance to other antibiotics, including aminoglycosides [e.g. *aac(6’)−29a*, *aph(3’’)-Ib*], beta-lactams (e.g. *bla*_TEM-1B_, *bla*_NDM-1_) and tetracyclines [e.g. *tet(B*), *tet(X4*)]. It is thus plausible that co-selection has played an important role in the dissemination of new macrolide resistance genes among proteobacterial pathogens.

The phylogenetic analysis revealed that macrolide resistance genes representing five of the six new pathogen-associated mobile gene families were found in species outside their indicated phylum of origin. This shows that inter-phyla transfer events of macrolide resistance genes do not only include a few widespread macrolide resistance genes, such as *erm(A*), *erm(B*) and *mph(B*), but also several new genes. Interestingly, genes from four of the new pathogen-associated mobile families were found located close to one or more genes encoding components of conjugation systems, including MOB relaxases, mating pair formation (MPF) genes, and type IV coupling proteins. The MOB genes found close to genes from UGF171 and UGF45 were of types that have a documented broad host range (P/Q according to the nomenclature of Smillie *et al*.) and have been previously reported in multiple phyla, including Proteobacteria, Firmicutes and Actinobacteria [54]. This suggests that conjugation enables the transfer of new macrolide resistance genes over very large evolutionary distances. Furthermore, genes from five of the pathogen-associated mobile gene families were found in the genetic vicinity of insertion sequences (ISs). Notably, IS*CR2* – a member of the IS*CR* family that uses a rolling circle transposition mechanism to move adjacent genes [55] – was found close to a total of 11 genes representing two new *erm* families and one new *mph* family (Table 3). IS*CR2* is one of the most widespread IS*CR*s and has previously been reported to be involved in the dissemination of multiple types of resistance genes, including macrolide phosphotransferases [56]. Since IS*CR2* was associated with genes from three of the six mobile gene families found in pathogens, it suggests that this MGE plays an important role in the dissemination of new macrolide resistance genes. However, IS*CR2* has, to our knowledge, not been identified outside of Proteobacteria to date, and indeed all occurrences identified in this study were in Proteobacteria. This would suggest that these new genes became associated with IS*CR2* after the initial inter-phyla transfer event and that other HGT mechanisms could have been responsible for their original mobilization. Furthermore, as the sequence identity shared between most of the new pathogen-associated mobile *erm* genes and their closest chromosomal genes in commensal or environmental bacteria was generally low (as low as 34.5% amino acid identity), this suggests either that the original mobilization of these genes happened in the ancient past or that the mobilization was more recent but the original host(s) are not represented in the databases. However, the *mph* family UGF5 of mobile genes in Enterobacteriaceae shared as much as 83.7% amino acid identity with chromosomal genes in Sphingobacteriaceae, indicating that this may be the result of a more recent transfer event.

Macrolide ARGs are present in bacteria from many phyla, however, their origin and evolutionary history have been largely unknown [57]. The phylogenetic analysis presented in this study provides a more detailed insight into their evolutionary history. For *erm* genes, we noted that the most widespread variants, including *erm(A*), *erm(B*) and *erm(C),* all clustered together into a single monophyletic clade together with species from the class Bacilli (cluster F.1, Fig. 3). Even though we were unable to identify their exact origin, it is clear that these genes share a close evolutionary relationship with Bacilli. Indeed, *erm(A*), *erm(B*) and *erm(C*) are all ubiquitously present in species from this class, especially in Bacillales, including *Staphylococcus* spp. [*erm(A*), *erm(C*)], and in Lactobacillales, including *Streptococcus* spp. [*erm(B*)]. Our phylogenetic analysis showed that all of these genes have undergone extensive HGT and they could be detected in both evolutionarily close and distant parts of the taxonomic tree (e.g. four, five and seven different phyla, respectively). Several of these transfer events have likely happened in the human or animal microbiome. For example, *erm(B*) had been transferred into a large number of pathogenic and non-pathogenic species from Clostridia (52), which, together with Bacilli, are ubiquitously present in the human and animal gut [58]. This indicates that the connectivity provided in the human microbiome, in combination with the presence of suitable MGEs and strong selection pressures caused by antibiotic consumption, may have favoured the mobilization and transfer of *erm* genes from their original hosts in Bacilli. We noted, however, that environmental Bacilli from the genus *Oceanobacillus* were also represented within cluster F.1. Though some species from this genus are known to colonize the human microbiome (e.g. *Oceanobacillus picturae *, *Oceanobacillus massiliensis *) [59, 60], an origin for *erm(A*), *erm(B*) and *erm(C*) outside the human and animal microbiome cannot be excluded.

In contrast, the most common *mph* genes [*mph(A*), *mph(B*), *mph(C*) and *mph(E*)] showed more diverse origins [12]. The phylogenetic analysis suggested that *mph(A*) originates from Proteobacteria, specifically Enterobacteriaceae, while *mph(B*) and *mph(C*) originate from Firmicutes (Clostridia and Bacilli, respectively). Finally, *mph(E*) was closely related to genes in Bacteriodetes, especially Sphingobacteriaceae, but was only found in one species from that phylum (* Myroides odoratimimus *), which likely reflects the under-representation of these genomes in the databases. Moreover, all of the most common *mph* genes, except for *mph(C*), have spread to several parts of the taxonomic tree, including many pathogens in Enterobacteriaceae. Indeed, *mph(A*) was, in addition to Proteobacteria, identified in Firmicutes (* Streptococcus suis *), while *mph(B*) and *mph(E*) were, in addition to their proposed original phyla, found in several proteobacterial species. We noted that *mph(C*), despite having a similar origin to *mph(B*) and being associated with MGEs, was not observed outside of Firmicutes. Our analysis of the genetic context showed that *mph(B*) genes were located on plasmids known to be associated with ARGs in Enterobacteriaceae (IncF, IncI, IncH) [61], likely explaining how they have been able to successfully spread among Proteobacteria. The association with an MGE with sufficient host range could thus explain why *mph(B*) has been able to successfully transfer to Proteobacteria when *mph(C*) has not. Indeed, previous studies have shown that *mph(C*) is fully functional when expressed in Enterobacteriaceae [62], suggesting that gene compatibility may not be a barrier. Nevertheless, our analysis underlines that *mph* genes are highly promiscuous and that they have been mobilized from several different phyla and transferred over large phylogenetic distances into human pathogens. This further demonstrates the need to characterize the full resistome, including the many *mph* genes that are present in distantly related species in order to understand their origin and evolutionary history.

Macrolides are naturally produced by several actinobacterial species, which carry a large diversity of ARGs, especially *erm* genes, that provide the means for self-resistance [63]. These genes are rarely transferred outside their phylum of origin and we, similarly to previous studies, found no indication that any of the common clinically relevant macrolide ARGs have been mobilized from Actinobacteria. However, contrary to some previous suggestions [64], our results show instances where macrolide resistance genes have successfully spread from antibiotic-producing Actinobacteria into Proteobacteria, proving that, while uncommon, it does occur. As an example, the new *erm* family UGF311, which was most closely related (41.2–44.3 % amino acid identity) to chromosomal genes found in the macrolide-producing Actinobacteria *Pseudonocardia* sp. and *Streptomyces venezuelae* [*erm(31*)] [3, 65], was identified on MGEs in *P. aeruginosa *. Similarly, the new *mph* family UGF100, which was most closely related (47.8–50.8% amino acid identity) to chromosomal genes in the actinobacterial family Micrococcaceae (especially *Zhihengliuella halotolerans *), was found in the Deltaproteobacteria *Myxococcus* spp. and *Corallococcus* spp. When expressed in *E. coli *, both these genes showed a significant impact on growth under a selection of erythromycin and azithromycin, demonstrating that these genes are compatible with proteobacterial hosts. UGF100 was commonly encountered in the predatory *Myxococcus xantus*, while UGF311 was flanked by IS elements relatively similar (67.3–86.1% amino acid identity) to ones associated with the predatory species *Cupriavidus necator* (IS*883*) [66]. Both *M. xanthus* and *C. necator* are known to prey on Actinobacteria and have recently been shown to be able to efficiently acquire actinobacterial genes (our data, unpublished). Based on these observations, we hypothesize that predatory bacteria may serve an important role in the inter-phyla transfer of ARGs in soil where they act as an intermediary between Actinobacteria and pathogenic Proteobacteria. It should, in this context, also be noted that transfers of macrolide resistance genes from Actinobacteria present in the human microbiome – such as *Bifidobacterium* spp., *Corynebacterium* spp. and *Brachybacterium* spp. – into Firmicutes and Proteobacteria have been reported but seems to be rare [e.g. *erm(X*) and *erm(50*)] [67]. Taken together, our results show that HGT of macrolide resistance genes from Actinobacteria to pathogens from other phyla is possible but limited. This is likely due to a combination of a multitude of barriers, such as the unavailability of MGEs with sufficient host range [54], missing ecological connectivity, and a lack of sufficiently strong selection pressures in some environments [18]. Thus, even though both commensal and environmental Actinobacteria carry a large and diverse macrolide resistome, they do not seem to yet have contributed significantly to the increasing macrolide resistance in human pathogens.

The genes identified in this study are based on computational predictions and should, until their function has been experimentally validated, be treated as putative macrolide resistance genes. Further analysis of the induced resistance phenotypes in pathogenic hosts beyond *E. coli* will be necessary to assess their full clinical relevance. We observed, however, a relatively even distribution of known, clinically relevant macrolide resistance determinants among the main clades found in the phylogenetic trees (Figs. 3 and 4) indicating that the predicted genes may confer a similar phenotype, unless their functionality has been recently lost. Indeed, it was noted that a certain degree of false positives was unavoidable when creating the Erm models, likely due to the close evolutionary relationship between *erm* genes and housekeeping methyltransferases of the KsgA family [11]. However, these *ksgA* genes were easily identified from their position in the phylogenetic tree (Fig. 3), and could thus be excluded from further consideration. Nonetheless, previous studies using the same methodology have displayed a high predictive power for other classes of resistance genes [20], and the experimental validation of selected new genes, where seven of ten gave resistance in *E. coli *, proves that our methodology could accurately predict functional new genes.

## Conclusions

Large-scale screening of sequence data resulted in more than 600 new families of *erm* and *mph* macrolide resistance genes – more than a tenfold increase compared to the genes known to date. Phylogenetic analysis indicated that *erm* genes have primarily been mobilized from the Firmicutes phylum while *mph* genes appear to have a more diverse origin with different variants from Firmicutes, Bacteroidetes and Proteobacteria. We identified several new mobile genes, including six previously uncharacterized genes present on MGEs in pathogenic bacteria of which four induced a resistance phenotype in *E. coli *. This study expands the knowledge about the macrolide resistome, including its diversity and evolutionary history. If mobilized and transferred into pathogens, these genes can threaten the efficacy of macrolides and thus, severely hamper our ability to treat bacterial infections. Our results also demonstrate that computational screening of bacterial genomes and metagenomes enables the detection of emerging resistance genes. This can potentially be used to implement new management strategies to prevent the global spread of novel forms of multi-drug-resistant bacteria.

## Supplementary Data

Supplementary material 1

Supplementary material 2

Supplementary material 2
