# Supplementary material for: Large-scale characterization of the macrolide resistome reveals high diversity and several new pathogen-associated genes
Source: Microb Genom. 2022 Jan 27;8(1):000770. doi: 10.1099/mgen.0.000770 (PMC8914350; doi:10.1099/mgen.0.000770)
Supplement: Supplementary material 1 [file mgen-8-0770-s001.pdf]

**Table S1.** List of the macrolide resistsance genes that were used to create the hidden Markov models.

| **Erm type A** | | **Erm type F** | | **Mph** | |
|---|---|---|---|---|---|
| **Protein name** | **Accession no.** | **Protein name** | **Accession no.** | **Protein name** | **Accession no.** |
| Erm(R) | AAA22075.1 | Erm(49) | OPG86592.1 | Mph(A) | BAA03770.2 |
| Erm(E) | CAB60001.1 | Erm(42) | CBY77552.1 | Mph(B) | BAA12910.1 |
| Erm(H) | AAC32026.1 | Erm(Q) | AAC36915.1 | Mph(C) | BAA34540.1 |
| Erm(V) | AAB51440.1 | Erm(47) | ANZ79475.1 | Mph(E) | CBY85745.1 |
| Erm(S) | AAA26742.1 | Erm(45) | CEJ95855.1 | Mph(F) | CAJ98570.1 |
| Erm(41) | ABW06859.1 | Erm(B) | AAA27452.2 | Mph(G) | BAL43359.1 |
| Erm(30) | AAC69328.1 | Erm(43) | CCF55073.1 | Mph(H) | WP_015776248.1 |
| Erm(W) | BAA03402.1 | Erm(T) | AAA98096.1 | Mph(I) | APB03226.1 |
| Erm(U) | WP_063844854.1 | Erm(A) | CAA26964.1 | Mph(J) | ATL63232.1 |
| Erm(31) | AAC69327.1 | Erm(F) | AAA98217.1 | Mph(K) | WP_003246254.1 |
| Erm(X) | AAA98484.1 | Erm(D) | AAA22599.1 | Mph(L) | EEL41021.1 |
| Erm(36) | AAL68827.1 | Erm(34) | AAP74657.1 | Mph(M) | WP_001041372.1 |
| Erm(46) | AJF36617.1 | | | Mph(N) | WP_024127776.1 |
| Erm(40) | AAS76623.1 | | | Mph(O) | WP_050815728.1 |
| Erm(N) | CAA66307.1 | | | | |
| Erm(Z) | CAM96571.1 | | | | |

**Table S2.** Sensitivity and specificity of the created profile HMs at the optimized threshold score.

| | | **Sensitivity** | | **Specificity** | |
|---|---|---|---|---|---|
| **Model** | **Reference sequences** | **Full-length** | **Reads (33AA)** | **Full-length** | **Reads (33AA)** |
| Erm type A | 16 | 0.9375 | 0.7668 | 1.000 | 0.9504 |
| Erm type F | 12 | 1.000 | 0.8058 | 1.000 | 0.9436 |
| Mph | 13 | 1.000 | 0.9663 | 1.000 | 0.9857 |

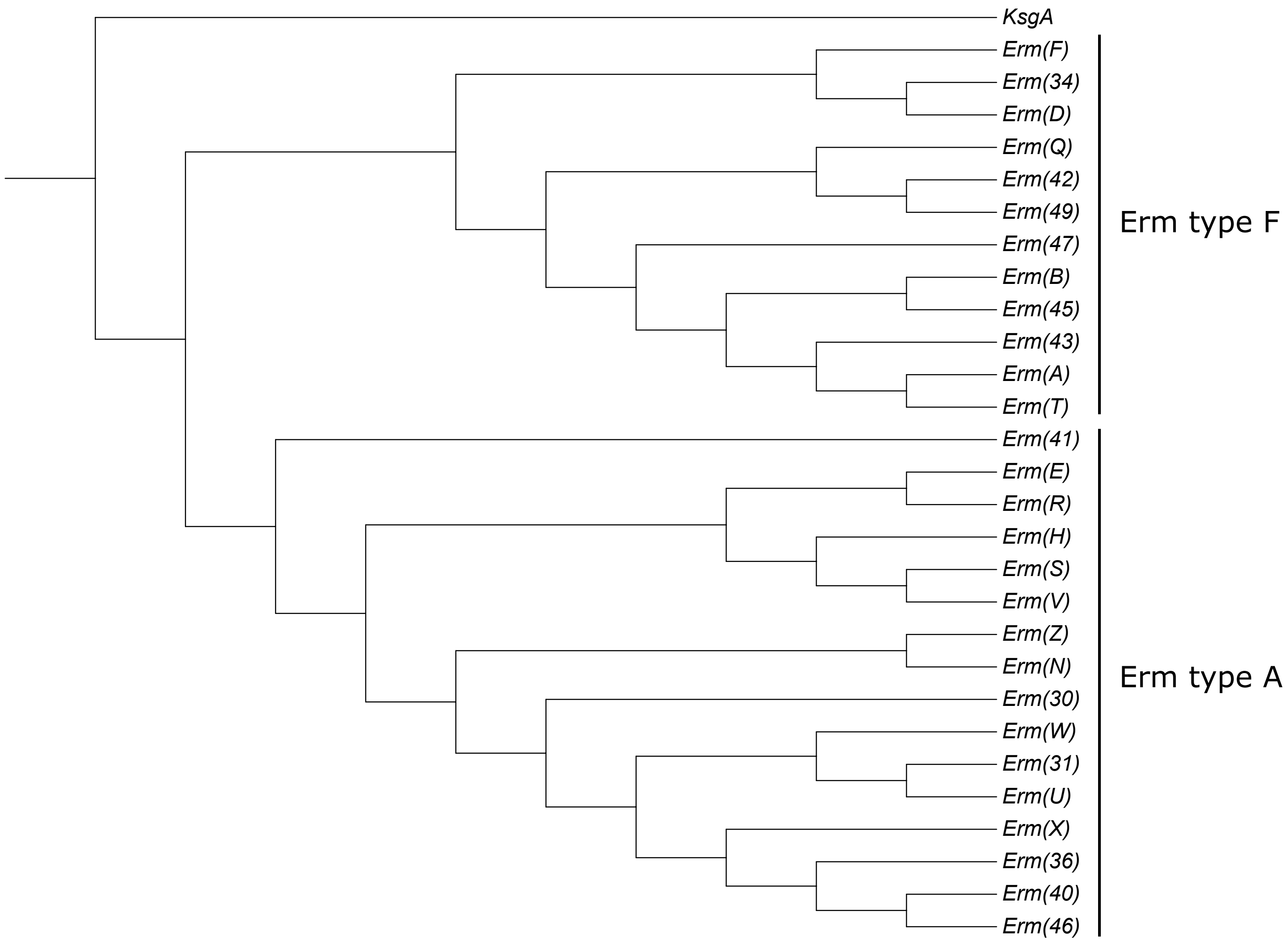


**Fig. S1.**
Dendrogram displaying known Erm sequences that displayed an AA identity of <70%. The two groups of sequences that were used to create separate models are marked in the tree.

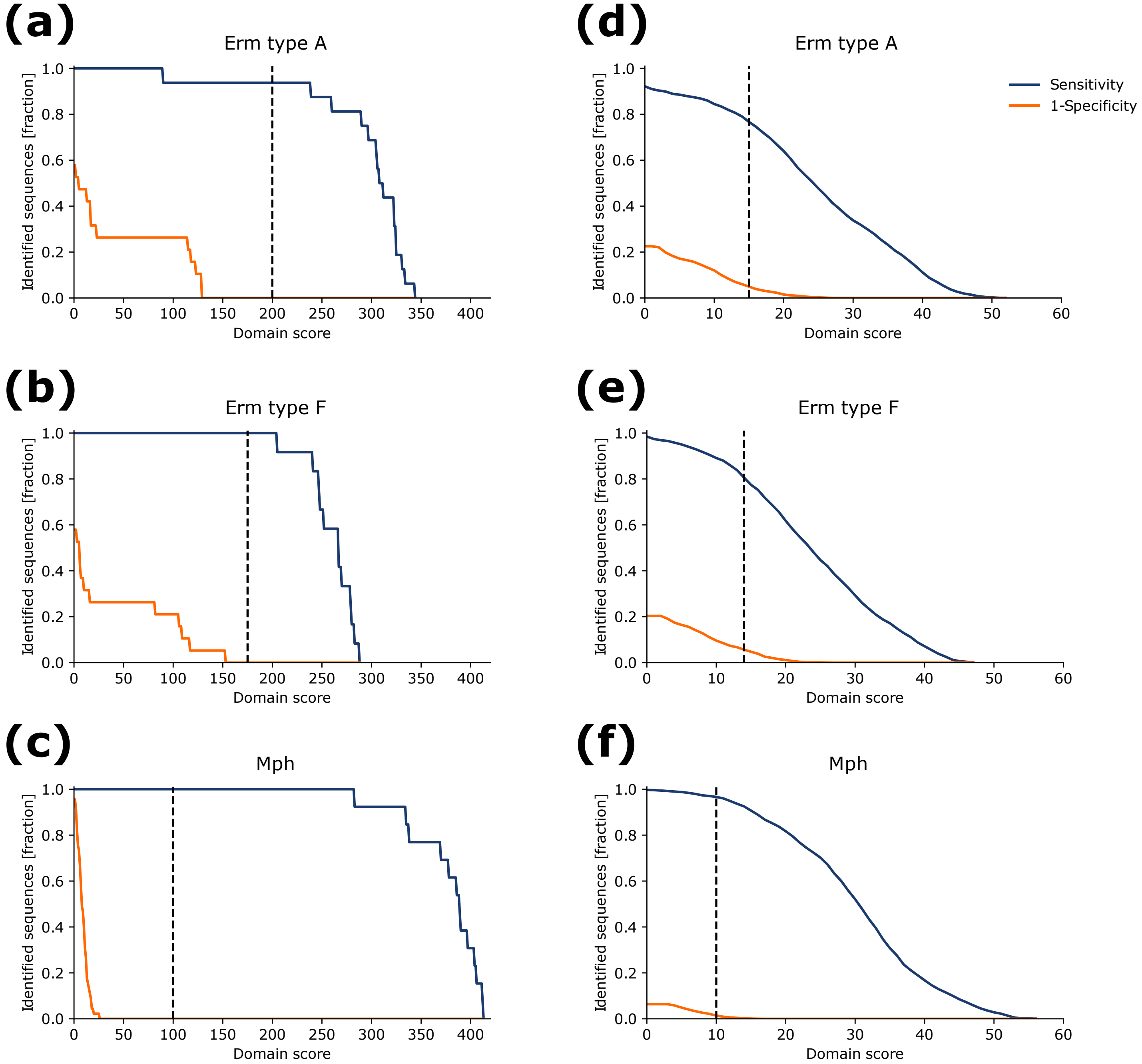


**Fig. S2.**
The sensitivity and specificity of the created HMMs as a function of domain score. Dashed black lines represent the optimized threshold scores used for analysis with the models. **(a)-(c):** Performance for classification of full-length genes for the three models. **(d)-(f):** Performance for classification of metagenomic fragments for the three models.

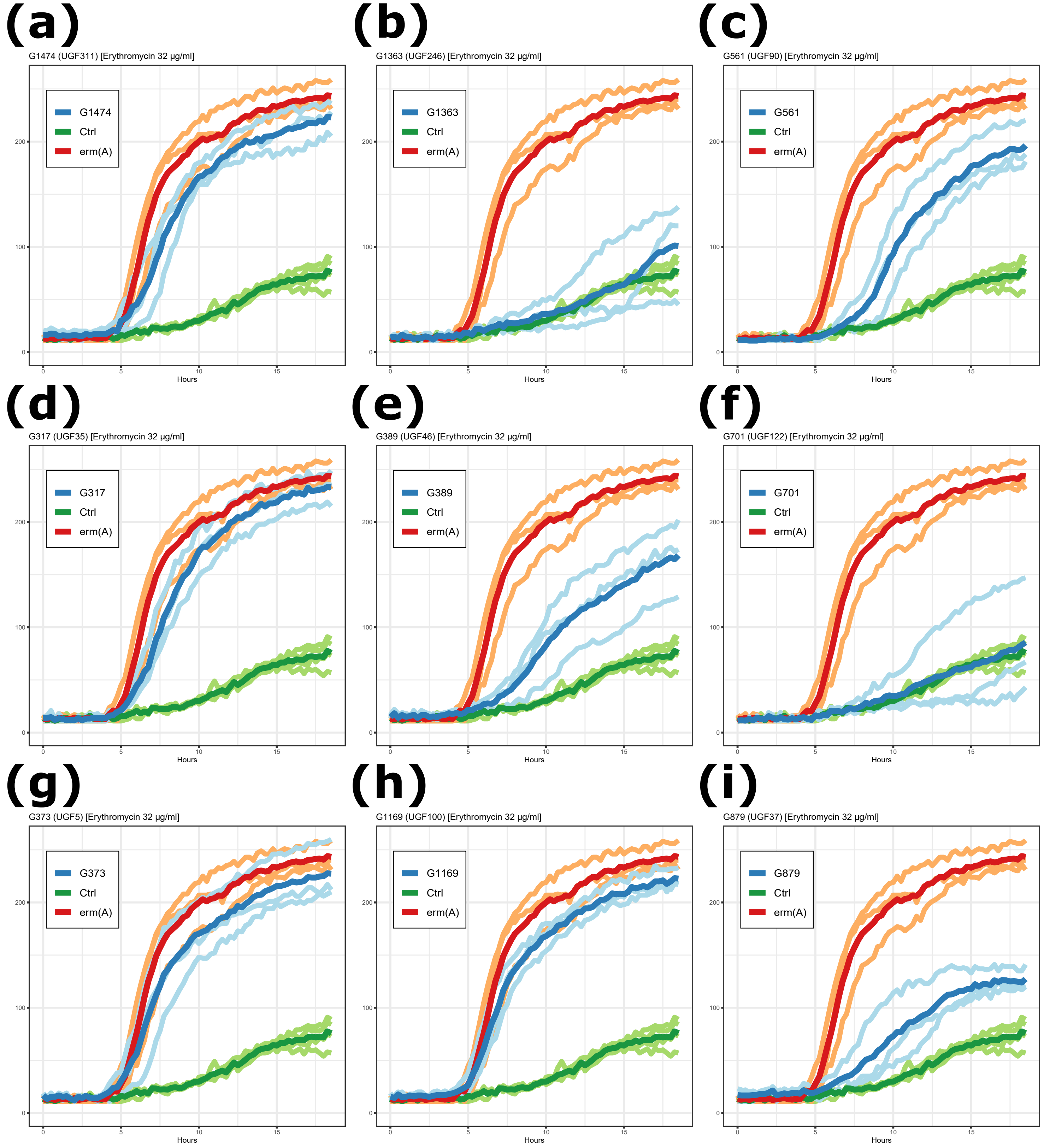


**Fig. S3.**
Growth behaviour of nine new macrolide resistance genes in *Escherichia coli* at 32µg/ml erythromycin compared to *erm(A)* and a negative control. In each group, lines with a stronger colour represent the mean of the measured replicates. **(a)-(f):** Growth curves of tested *erm* genes (G1474 (UGF311), G1363 (UGF246), G561 (UGF90), G317 (UGF35), G389 (UGF46), G701 (UGF122)). **(g)-(i):** Growth curves of the tested *mph* genes (G373 (UGF5), G1169 (UGF100), G879 (UGF37)).

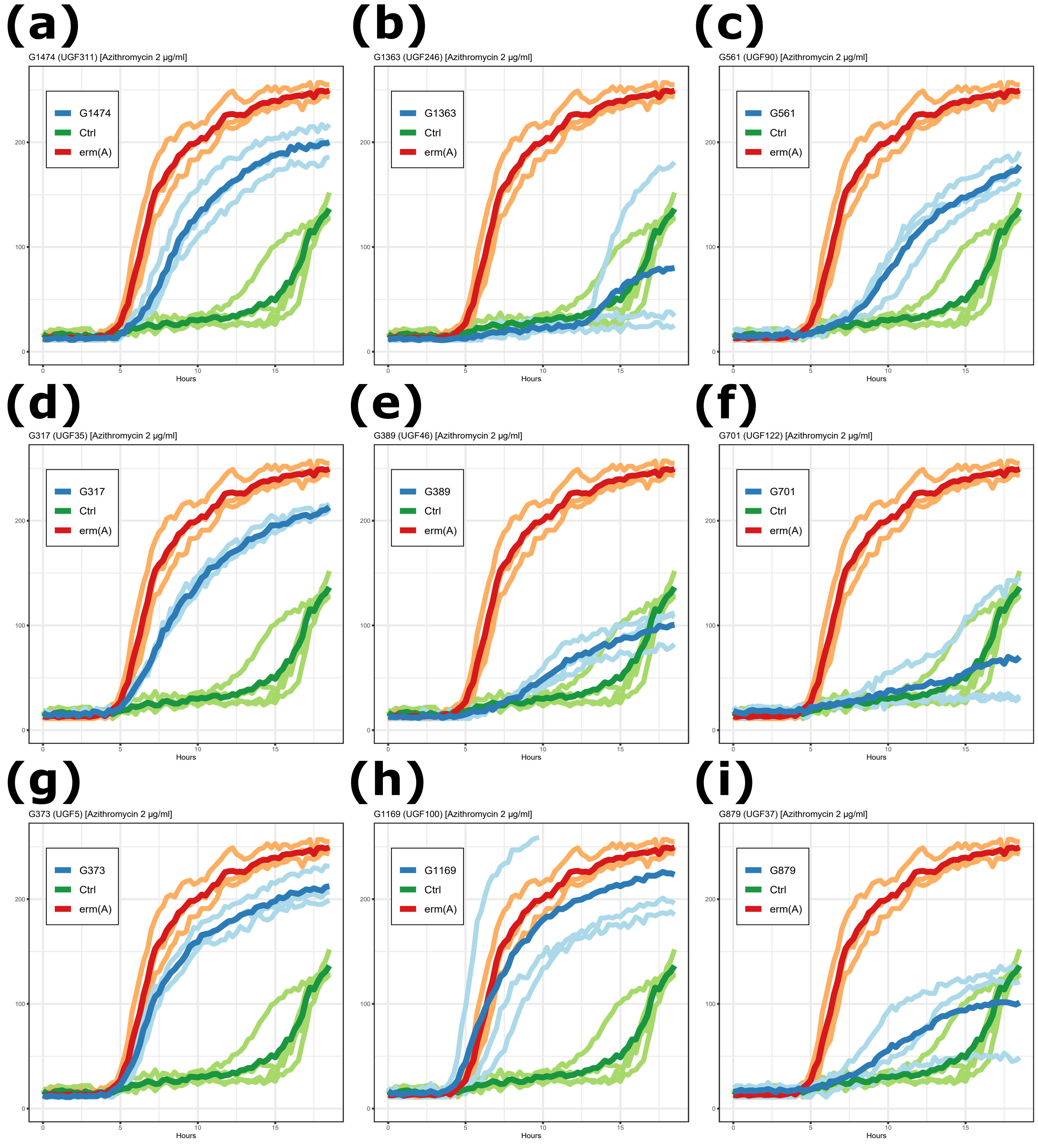


**Fig. S4.**

Growth behaviour of nine new macrolide resistance genes in *Escherichia coli* at 2µg/ml azithromycin compared to *erm(A)* and a negative control. In each group, lines with a stronger colour represent the mean of the measured replicates. **(a)-(f):** Growth curves of tested *erm* genes (G1474 (UGF311), G1363 (UGF246), G561 (UGF90), G317 (UGF35), G389 (UGF46), G701 (UGF122)). **(g)-(i):** Growth curves of the tested *mph* genes (G373 (UGF5), G1169 (UGF100), G879 (UGF37)).

**Fig. S5.**
Phylogenetic tree depicting the Erm 23S rRNA methyltransferases predicted in this study.

**Fig. S6.**
Phylogenetic tree depicting the Mph macrolide 2'-phosphotransferases predicted in this study.

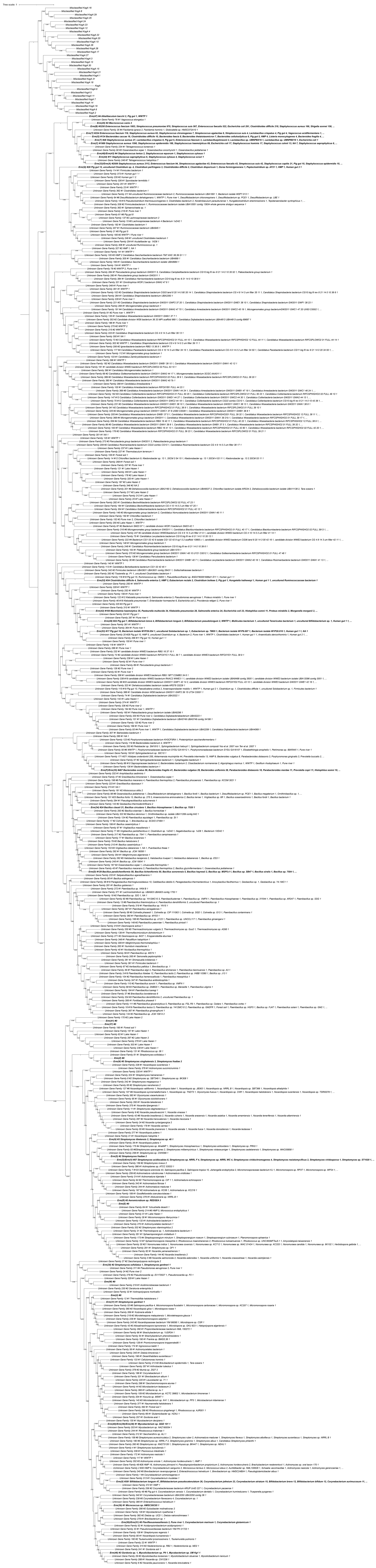
Tree scale: 1

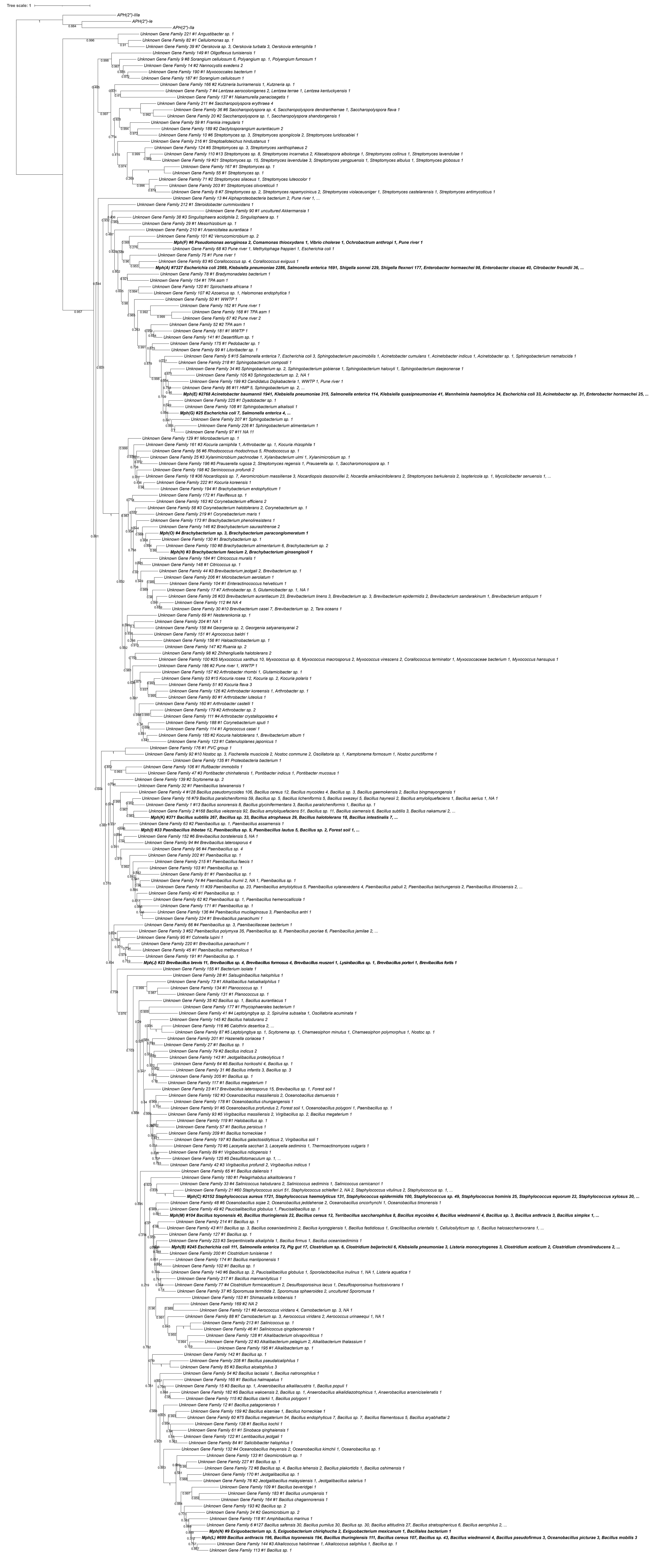
Tree scale: 1
APH(2")-IIIa
APH(2")-Ie
APH(2")-IIa
